# Efficacy and safety of fenofibrate add-on therapy in patients with primary biliary cholangitis refractory to ursodeoxycholic acid: A retrospective study and updated meta-analysis

**DOI:** 10.3389/fphar.2022.948362

**Published:** 2022-08-30

**Authors:** Xuan Guoyun, Ding Dawei, Liu Ning, Hu Yinan, Yang Fangfang, Tian Siyuan, Sun Hao, Yang Jiaqi, Xu Ang, Guo Guanya, Chen Xi, Shang Yulong, Han Ying

**Affiliations:** ^1^ National Clinical Research Centre for Digestive Diseases, State Key Laboratory of Cancer Biology, Xijing Hospital of Digestive Diseases, Air Force Medical University (Fourth Military Medical University), Xi’an, China; ^2^ Medical Service, The Air Force Hospital of Southern Theater of PLA, Guangzhou, China

**Keywords:** primary biliary cholangitis, monotherapy, meta-analysis, clinical trial, fenofibrate

## Abstract

**Background:** Ursodeoxycholic acid (UDCA) is currently used for the treatment of primary biliary cholangitis (PBC), but some people do not respond well to UDCA. It reported that the combination of fenofibrate and UDCA can improve the clinical indices in these patients. However, more high-quality evidence is needed to improve guideline recommendations.

**Methods:** Through an updated meta-analysis, studies included were valued by the Cochrane Evaluation Manual and Robins-I. Biochemical and clinical indicator changes in UDCA-refractory PBC patients receiving combination therapy were analyzed by Revman 5.42. Then, we explored the influence of fenofibrate dose and the effectiveness and safety of long-term application by retrospective cohort study.

**Results:** Our meta-analysis included nine publications with a total of 389 patients, including 216 treated with UDCA alone and 173 who received combination therapy. The meta-analysis showed that combination therapy was more effective than UDCA monotherapy in decreasing biochemical parameters, such as ALP, GGT, IgM, and TG. However, the occurrence of pruritus and adverse events was slightly higher with combination therapy than with UDCA monotherapy. A total of 156 patients were included in our cohort study: 68 patients underwent UDCA monotherapy, and 88 patients underwent combination therapy. Among UDCA-refractory patients, fenofibrate add-on therapy significantly improved the ALP normalization rate.

**Conclusion:** The combination of fenofibrate and UDCA can decrease biochemical parameters, of UDCA-refractory PBC patient. Furthermore, the efficacy and safety of long-term combination therapy were also confirmed in our cohort study.

## Introduction

Primary biliary cholangitis (PBC), also known as primary biliary cirrhosis, is a chronic autoimmune intrahepatic cholestatic disease (Lleo et al., 2020). Its pathogenesis is not fully understood, but it may be related to abnormal autoimmune responses caused by the interaction of genetic background (Olafsson et al., 2004) and environmental factors (Matsumoto et al., 2022). PBC is mainly observed in middle-aged and elderly women, and the most common presenting symptoms are fatigue and skin pruritus. Serum antimitochondrial antibody (AMA) positivity, especially the positive AMA-M2 subtype, has high sensitivity and specificity for the diagnosis of PBC (Zandanell et al., 2021). At present, ursodeoxycholic acid (UDCA) is still the only drug that has been proven safe and effective in the treatment of PBC by randomized controlled clinical trials (RCT). UDCA can improve biochemical parameters in PBC patients. Several randomized controlled studies and meta-analyses have shown that UDCA can effectively reduce serum total bilirubin (TBIL), alkaline phosphatase (ALP), gamma-glutamyltransferase (GGT), alanine aminotransferase (ALT), aspartate aminotransferase (AST) and cholesterol (CHO) levels (Dat et al., 2021). There are several international criteria for evaluating biochemical response after UDCA treatment (Lammers et al., 2014). Among those criteria, the Paris I and Paris II criteria are frequently used to evaluate biochemical responses to UDCA in patients with advanced PBC (stage III-IV) and early PBC (stage I-II), respectively (Corpechot et al., 2008). Patients with adequate biochemical response to UDCA have a greatly improved survival rate (Harms et al., 2019). However, approximately 40% of patients with PBC have inadequate biochemical response to UDCA monotherapy, so we define them as UDCA-refractory PBC patients. And there is a significant reduction in long-term survival for this group of patients, which is a problem for clinical treatment at present.

There is currently no unified treatment for UDCA-refractory PBC patients (Hirschfield et al., 2021). Scholars from Japan, the United States, Europe and China have successively reported the application of fenofibrate in UDCA-refractory PBC patients (Dohmen et al., 2004; Han et al., 2012; Liberopoulos et al., 2010; Levy et al., 2011; Ohira et al., 2002). A meta-analysis published in 2015 showed that the combination of fenofibrate and UDCA decreased the levels of ALP, GGT, immunoglobulin M (IgM) and triglyceride (TG) compared with UDCA monotherapy, but there was no significant difference in the improvement of skin pruritus or ALT. In addition, there was no significant difference in the occurrence of adverse events between combination therapy and monotherapy. Whether fenofibrate can improve the long-term outcomes of patients with PBC is unclear (Zhang et al., 2015).

Although fenofibrate has been recommended, the guidelines of various countries and regions do not explicitly recommend the dosage of fenofibrate, and there is no relevant research report on the dosage of fenofibrate (Chinese Society of Hepatology and Chinese Medical Association, 2022; European Association for the Study of the Liver, 2017; Lindor et al., 2018), the meta-analysis is still needed to provide medical evidence. A recent related meta-analysis was the work of Zhang et al., in 2015 (Zhang et al., 2015). Their work was based on the fact that the quality of clinical studies included in their paper needs to be improved. Furthermore, some relevant clinical studies (Cheung et al., 2016; Duan et al., 2018; Hegade et al., 2016) and new evaluation criteria including Robins-I (Sterne et al., 2016) have emerged since 2015. To this end, we try to include higher quality studies, and attempt to carry out subgroup analysis on fenofibrate dose and integrate retrospective cohort study of our center to give more specific opinions for clinical practice.

## Methods

### Identification of studies for inclusion in the meta-analysis

This meta-analysis was registered in INPLASY (registration no. INPLASY202230116). The included studies were identified in English databases, including PubMed, Embase, and The Cochrane Library (updated to December 2021), by a manual search for relevant literature using the search terms “ursodeoxycholic acid”, “UDCA”, “fenofibrate”, “PBC”, “primary biliary cholangitis”, “primary biliary cirrhosis” and “randomized controlled trial”. Further literature was searched to prevent omission.

### Inclusion and exclusion criteria

Studies included in this study met the following five criteria. 1) Randomized controlled trial or clinical controlled trial comparing combination therapy and UDCA monotherapy. 2) PBC was diagnosed when any two of the following three criteria are met: 1) biochemical evidence of cholestasis (ALP and GGT) is present, and imaging excludes extrahepatic or intrahepatic bold duct obstruction; 2) positive for AMPA/AMA⁃M2 or other PBC-specific autoantibodies (anti-gp210 antibodies and anti-sp100 antibodies); and 3) histological evidence of nonsuppurative destructive cholangitis and small bile duct disruption. 3) Complete biochemical response to treatment is defined as a decrease in ALP level of more than 40% of the baseline value or ALP level in the normal range after 1 year of UDCA treatment. 4) All patients were not treated with other liver disease medications. 5) For a study produced by the same team, the results with the largest number of cases and most complete data were taken. The studies excluded in this study met the following two criteria. 1) Duplicate documents. 2) Literature with no data to extract.

### Data extraction and assessment of risk of bias

The following data were extracted from each included article: name of the first author, date of publication, sex, age, number of patients, treatment dose and duration, biochemical indices, clinical symptoms, adverse effects, survival rate, etc. Data were independently collected from each study by two researchers (Guoyun Xuan and Ning Liu) to confirm the accuracy of the data. The included studies were evaluated by the researchers according to the Cochrane Evaluation Manual based on the following six aspects: 1. Random assignment method. 2. Concealment of the assignment scheme. 3. Use of blinding. 4. Completeness of the outcome data. 5. Selective reporting of outcomes. 6. Other sources of bias. The risk of bias was also checked independently by the researchers, with answers “Yes” indicating low risk of bias, “No” indicating high risk of bias, and “Unclear” indicating either a lack of information or uncertainty over the potential for bias. Cochrane Reviews often include RCT. Therefore, risk of bias should be assessed for each included study by Robins-I (Lindor et al., 2018). In the current study, the researchers evaluated the included studies based on the following seven dimensions: 1. Bias due to confounding; 2. Bias in selection of participants into the study. 3. Bias in classification of interventions. 4. Bias due to deviations from intended interventions. 5. Bias due to missing data. 6. Bias in measurement of the outcome. 7. Bias in selection of the reported result. The risk of bias in evaluating non-randomized controlled trials was all examined independently by the investigators, and the evaluation results were classified as: “+” indicating low risk of bias, “?” indicating moderate risk of bias, “-” indicating serious risk of bias.

### Clinical study design

A retrospective cohort study was conducted. Patients were divided into “the UDCA group” and “the UDCA + FF group” depending on whether they received treatment with UDCA monotherapy or combination therapy of fenofibrate and UDCA. We explored the efficacy and safety of fenofibrate add-on therapy by comparing the clinical characteristics of the two groups. We systematically collected clinical information at presentation and each follow-up. Data included general characteristics, clinical symptoms and serology results. Biochemical response was determined by achieving normal serum ALP levels during follow-up. To evaluate the efficacy of fenofibrate, the primary outcome was the percentage of cases with biochemical responses. Hepatic deterioration was determined by the presence of a decompensatory event (such as hepatic encephalopathy, ascites, or variceal bleeding) and/or progression of the Child-Pugh grade by at least one level (Arroyo et al., 1996). The safety of fenofibrate was assessed primarily in terms of fenofibrate-related symptoms (Levy et al., 2011), hepatotoxicity and nephrotoxicity (detailed characteristics can be seen in Supplementary Table S1). The Chronic Kidney Disease Epidemiology Collaboration (CKD-EPI) equation was used to calculate eGFR values. The study design was approved by the ethics committee of the Xijing Hospital of the Air Force Military Medical University (KY20151230-5).

### Study population

We analyzed 156 consecutive subjects with PBC who were refractory to prior UDCA monotherapy for 6 months diagnosed and treated in Xijing Hospital of Digestive Diseases (Xi’an, Shaanxi, China) from February 2010 to November 2020. Patients with evidence of concomitant liver disease (autoimmune hepatitis, alcoholic hepatitis, drug-induced liver injury, viral hepatitis, hemochromatosis, and hepatocellular carcinoma) were excluded from our design. A diagnosis of PBC was made if the case met two of the three standards mentioned above. The definition of refractory to UDCA was failure to meet the ALP cutoff value (serum ALP >1.67x ULN) utilized in the Toronto criteria (Corpechot et al., 2011) after 6 months of prior UDCA monotherapy. UDCA and fenofibrate were administered orally at doses of 13–15 mg/kg/d and 200 mg/d, respectively.

### Statistical analysis

In the meta-analysis, the extracted data were processed using the Cochrane systematic evaluation software Revman 5.42 (The Nordic Cochrane Centre, Copenhagen, Denmark; The Cochrane Collaboration, 2012). For dichotomous outcomes, the odds ratio (OR) value and 95% confidence interval (95% CI) were calculated. For continuous variable outcomes, the mean difference (MD) and 95% confidence intervals (CIs) were used. We mainly used the χ2 test and the *I* ^*2*^ test to test heterogeneity (*p* value <0.10 or *I* ^*2*^ value >50% was considered significant heterogeneity), and then random effect model analysis was performed. Fixed-effects models were used (*p* value >0.10 and *I* ^*2*^ value <50%). Subgroup and sensitivity analyses were performed. In our clinical study, SPSS version 26.0 (SPSS Inc., Chicago, IL, United States) was used for all statistical analyses. Continuous variables were described as mean and standard deviation (SD), whereas categorical variables were expressed as median and interquartile range. Categorical data were compared using χ2 or Fisher’s exact test where appropriate, whereas the Mann-Whitney *U* test was used to analyze continuous non-normally distributed variables. Comparisons between biochemical variables at baseline and after treatments were performed using the Wilcoxon signed-rank test for paired date. A two-sided *p* < 0.05 was considered statistically significant.

## Results

### Description and qualitative assessments of meta-analysis

**FIGURE 1:**
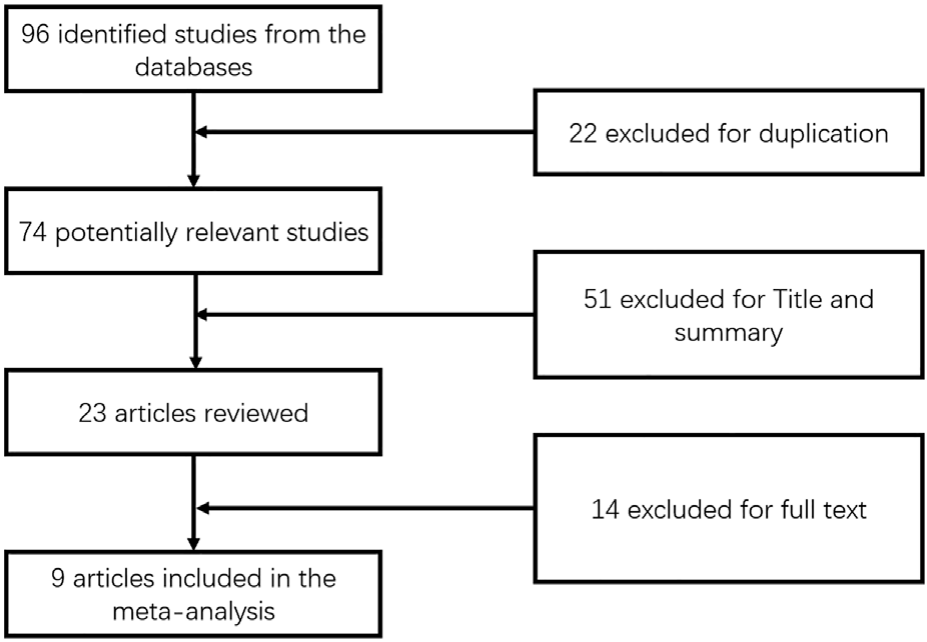
Flow diagram of the studies included in the meta-analysis.

This meta-analysis included nine publications (Cheung et al., 2016; Dohmen et al., 2004; Duan et al., 2018; Han et al., 2012; Hegade et al., 2016; Liberopoulos et al., 2010; Levy et al., 2011; Ohira et al., 2002; Walker et al., 2009) published from 2002 to 2021, including a total of 389 patients (Figure 1) aged 51–61 years and a follow-up period of 3–24 months. The daily dose of UDCA ranged from 13 to 15 mg/kg day, and the daily dose of fenofibrate was 100–200 mg/day. (Table 1).

**TABLE 1:** Baseline characteristics of the trials included in the meta-analysis.

References	Publication date	Mean age (years)	UDCA dose (mg/day)	Fenofibrate dose (mg/day)	UDCA		UDCA + FF	
					Course of treatment	Patiens(n)	Course of treatment	Patiens(n)
Ohira, H. Ohira et al. (2002)	2002	61	600–900	150–200	8 years	7	6 months	7
Dohmen, K. Dohmen et al. (2004)	2004	53	600	100–150	6 months	9	3 months	9
Walker, L. J. Walker et al. (2009)	2009	55	600–900	134–200	23 months	16	23 months	16
Liberopoulos, E. N. Liberopoulos et al. (2010)	2010	57	600	200	8 months	6	2 months	4
Levy, C. Levy et al. (2011)	2011	56	600–900	160	12 months	20	24 months	20
Han, X. F. Han et al. (2012)	2012	51	13–15 mg/kg/day	200	18 months	22	3 months	22
Cheung A C Cheung et al. (2016)	2016	53	600–900	145	12 months	74	11 months	46
Hegade, V. S. Hegade et al. (2016)	2016	56	600–900	200	12 months	23	12 months	23
Duan Weijia Duan et al. (2018)	2018	54	600–900	200	12 months	39	24 months	26

Abbreviations: UDCA, ursodeoxycholic acid; COM, the combination therapy of fenofibrate and UDCA.

**FIGURE 2:**
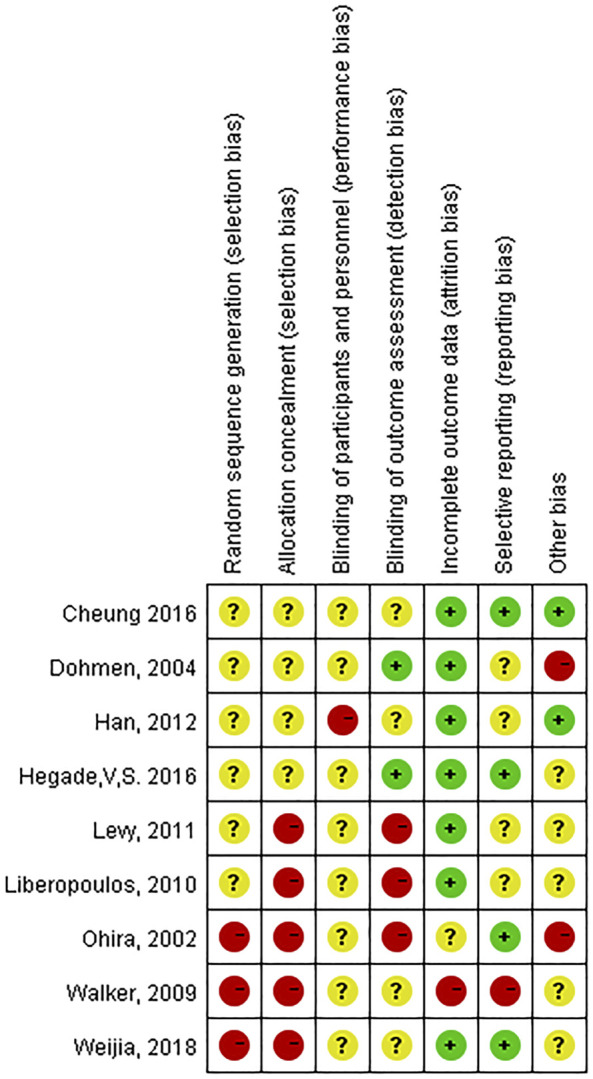
Risk of bias in the included studies.

**FIGURE 3:**
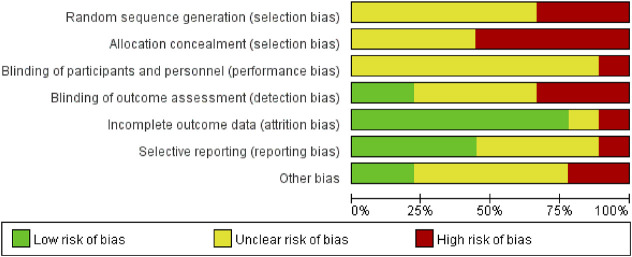
Risk of bias graph: review of the authors’ judgements regrading each risk of bias item presented as percentages across all included studies.

**FIGURE 4:**
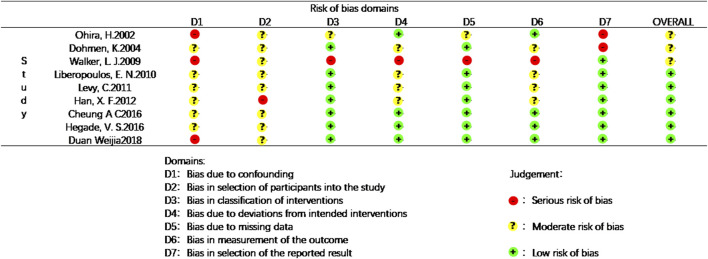
Risk of bias in the included studies based on Robins-I.

Data for the nine studies are shown in Table 2. The nine studies included were also evaluated for quality. Figure 2 shows the evaluation of all included literature relative to each bias risk item. Figure 3 shows the risk of bias summary plot, which is an assessment of the percentage of risk of bias items arising from all included literature. Those studies include non-randomized studies of interventions (NRSI) as shown in Figure 3. Therefore, risk of bias was assessed for each included study by Robins-I in Figure 4 (Lindor et al., 2018).

**TABLE 2:** Meta-analysis of clinical events and biochemical parameter changes in the included studies.

Outcome title	Number of studies	Number of participants	Statistical method	Effect size	*p*-value	I2 (%)
Alkaline phosphatase level	9	389	Mean difference (IV, Fixed, 95% CI)	-106.29 [-132.56, -80.02]	*p* < 0.00001	48
Gamma-glutamyl transferase	4	86	Mean difference (IV, Fixed, 95% CI)	-78.57 [-135.42, -21.72]	*p* = 0.007	6
Alanine aminotransferase	5	232	Mean difference (IV, Fixed, 95% CI)	-5.40 [-14.56, 3.76]	*p* = 0.25	42
Aspartate aminotransferase	5	232	Mean difference (IV, Fixed, 95% CI)	-4.89 [-11.65, 1.87]	*p* = 0.16	48
Immunoglobulin M	5	114	Mean difference (IV, Fixed, 95% CI)	-80.24 [-93.46, -67.02]	*p* < 0.00001	0
Triglycerides	4	112	Mean difference (IV, Fixed, 95% CI)	-0.38 [-0.55, -0.21]	*p* < 0.0001	40
Total bilirubin	4	112	Mean difference (IV, Fixed, 95% CI)	-0.44 [-3.12, 2.24]	*p* = 0.75	0
Creatinine	4	241	Mean difference (IV, Fixed, 95% CI)	9.77 [4.05, 15.49]	*p* = 0.0008	34
Pruritus	5	228	Odds ratio (M–H, Fixed, 95% CI)	4.08 [1.10, 15.16]	*p* = 0.04	0
Adverse events	6	246	Odds ratio (M–H, Fixed, 96% CI)	13.44 [1.70, 105.98]	*p* = 0.01	0

Abbreviations: IV, inverse-variance; CI, confidence interval; M-H, Mantel-Haenszel methods; fixed, fixed effects model.

### Meta-analysis of biochemical response and adverse events

The results (Table 2) showed that the effect of the combination group in decreasing ALP (MD: 98.08 IU/L, 95% CI: 110.11 to - 86.06, *p* < 0.00001) (Supplementary Figure S1), GGT (MD: 78.57 IU/L, 95% CI: 135.42 to - 21.72, *p* = 0.007) (Supplementary Figure S2), IgM (MD: 80.24 mg/dl, 95% CI: 93.46 to - 67.02, *p* < 0.00001) (Supplementary Figure S5), TG (MD: 0.38 mg/dl, 95% CI: 0.55 to - 0.21, *p* < 0.0001) (Supplementary Figure S6) and other indices were significantly better than those of the UDCA monotherapy group, but there was no statistical significance in the improvement in ALT (MD: 5.40 IU/L, 95% CI: 14.56 to 3.76, P: 0.25) (Supplementary Figure S3), AST (MD: 4.89 IU/L, 95% CI: 11.65 to 1.87, P: 0.16) (Supplementary Figure S4) or TBIL (MD: 0.44 IU/L, 95% CI: 3.12 to 2.24, P: 0.75) (Supplementary Figure S7) level. The incidence of pruritus and adverse events in the combined group were higher than those of the single drug treatment group, but the differences were not obvious [pruritus (MD: 4.08, 95% CI: 1.10 to 15.16, P: 0.04) (Supplementary Figure S9); adverse events (MD: 13.44, 95% CI: 1.70 to 105.98, P: 0.01) (Supplementary Figure S10)]. The effect of the combined treatment on creatinine (CRE) level was not statistically significant (MD: 9.77 IU/L, 95% CI: 4.05 to 15.49, *p* = 0.0008) (Supplementary Figure S8).

**FIGURE 5:**
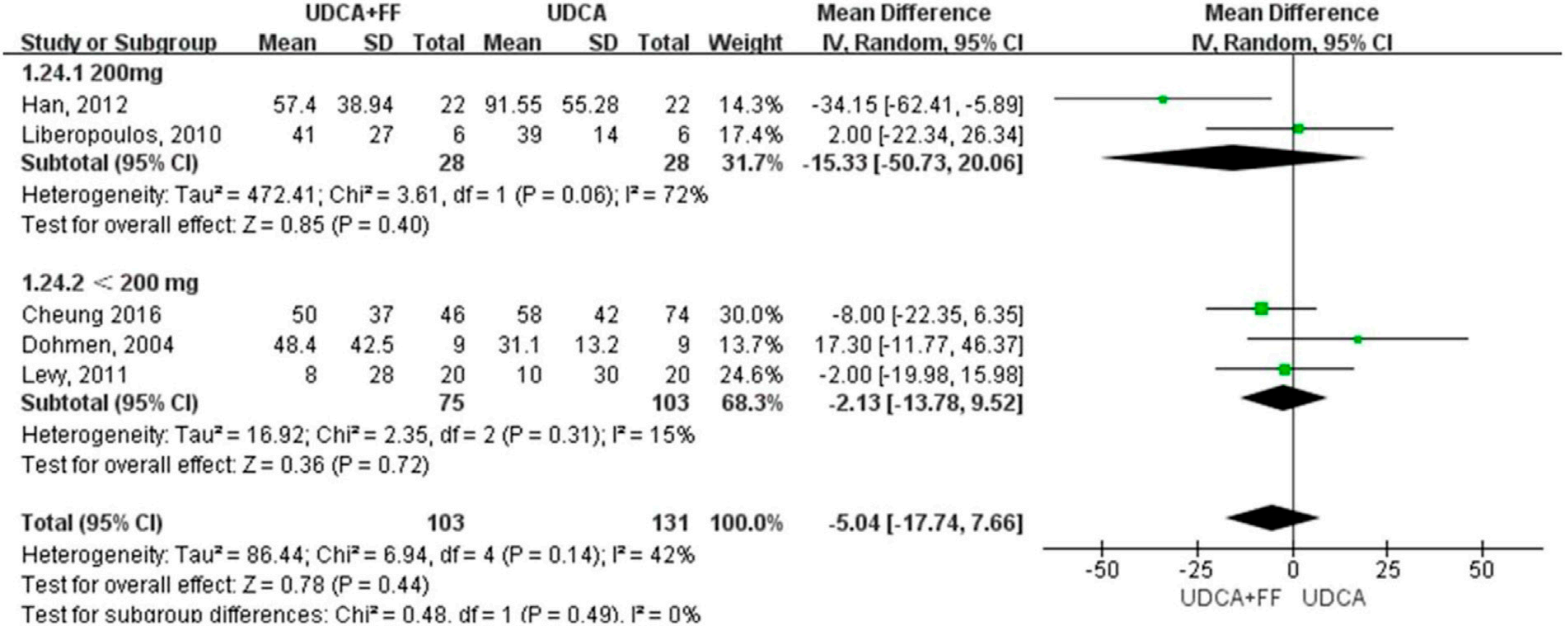
Subgroup analysis of ALP levels in PBC patients treated with monotherapy versus COM.

**FIGURE 6:**
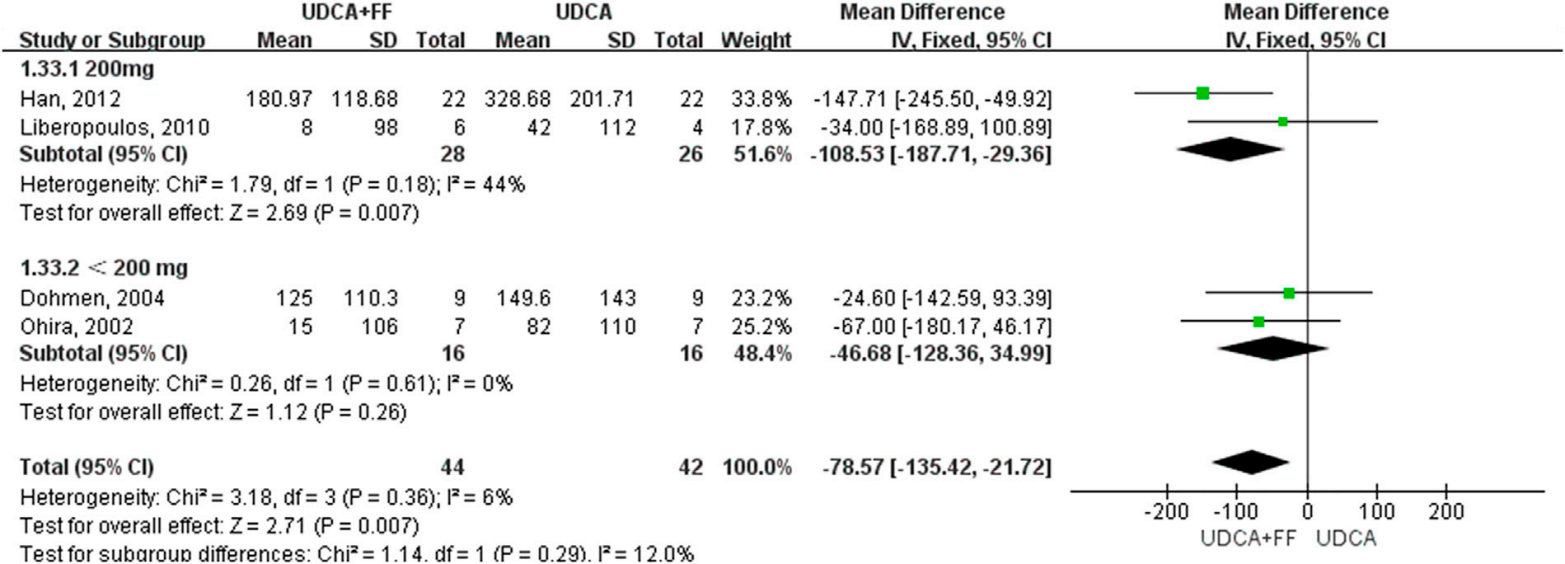
Subgroup analysis of GGT levels in PBC patients treated with monotherapy versus COM.

We further grouped the studies according to the dosage of fenofibrate: less than 200 mg/day and 200 mg/day. The subgroup analysis based on identified prognostic indicators was showed in Figure 5 and Figure 6. Although not statistically significant, the results showed that 200 mg per day fenofibrate might achieve stable therapeutic effect in reducing ALP and GGT (Figure 5 and Figure 6).

### Clinical features of patients in the UDCA + FF group and the UDCA group

**FIGURE 7:**
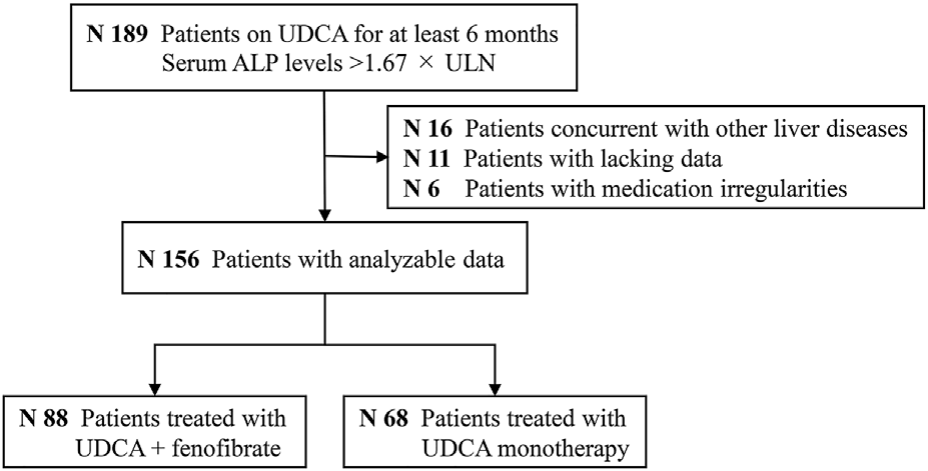
Study flowchart. Abbreviations: ALP, alkaline phosphatase; UDCA, ursodeoxycholic acid; FF, fenofibrate.

Figure 7 is the cohort study flowchart. Of the 156 patients with PBC refractory to UDCA, 88 (56%) were treated with combination therapy of fenofibrate and UDCA (UDCA + FF group), and 68 (44%) continued with UDCA monotherapy (the UDCA group). Fenofibrate was administered on average 18 ± 12 months after the start of UDCA. The mean time of exposure to fenofibrate was 42 ± 29 months. No significant differences except for serum GGT, IgG, and TG levels were identified for either group at baseline (*p* < 0.001, all) (Table 3). Liver histology data on the stage of fibrosis (results of the last liver biopsy at the time of enrollment) were available in 145 (93%) patients. Depending on the histological findings, a total of 25% of the patients were in advanced stages of disease (Ludwig stage 3 or 4).

**TABLE 3:** Baseline characteristics of patients with UDCA-refractory primary biliary cholangitis treated with “UDCA + FF” or “UDCA monotherapy”.

Characteristic	COM group	UDCA group	*p* value
	N = 88	N = 68	
Age (mean ± SD)	50 ± 10	52 ± 8	*p* = 0.424
Female (n, %)	71 (81)	61 (90)	*p* = 0.121
Follow-up Time (months)	42 ± 26	43 ± 26	*p* = 0.829
ALP×ULN	2.5 (2.0–3.3)	2.4 (2.0–3.3)	*p* = 0.677
GGT×ULN	7.9 (4.6–13.0)	5.3 (3.2–8.0)	*p* < 0.001
ALT×ULN	1.7 (1.2–2.3)	1.5 (1.0–2.3)	*p* = 0.271
AST×ULN	1.9 (1.4–2.5)	2.0 (1.4–2.7)	*p* = 0.694
ALB×LLN	1.1 (1.0–1.1)	1.0 (1.0–1.1)	*p* = 0.203
Tbil×ULN	1.0 (0.8–1.6)	1.0 (0.7–1.5)	*p* = 0.790
IgG×ULN	0.8 (0.7–0.9)	0.9 (0.8–1.1)	*p* < 0.001
IgM×ULN	1.2 (0.8–1.8)	1.4 (0.8–1.9)	*p* = 0.432
TG×LLN	1.0 (0.7–1.5)	0.8 (0.6–1.0)	*p* < 0.001
BU×ULN	0.6 (0.5–0.7)	0.6 (0.5–0.7)	*p* = 0.109
Scr×ULN	1.0 (0.9–1.1)	1.0 (0.9–1.1)	*p* = 0.656
eGFR (ml/min/1.73m2)	97 (91–102)	95 (89–102)	*p* = 0.216
Fibrosis stage (0–2)/(3–4)	62/21	45/18	*p* = 0.690

Abbreviations: ALP, alkaline phosphatase; GGT, gamma-glutamyl transferase; ALT, alanine-aminotransferase; AST, aspartate-aminotransferase; ALB, albumin; Tbil, total bilirubin; IgG, immunoglobulin G; IgM, immunoglobulin M; TG, triglyceride; BU, blood urea; Scr, serum creatinine; eGFR, estimated glomerular filtration rate; ULN, upper limit of normal; LLN, lower limit of normal; FF, fenofibrate; UDCA, ursodeoxycholic acid.

### Primary and secondary outcomes

**FIGURE 8:**
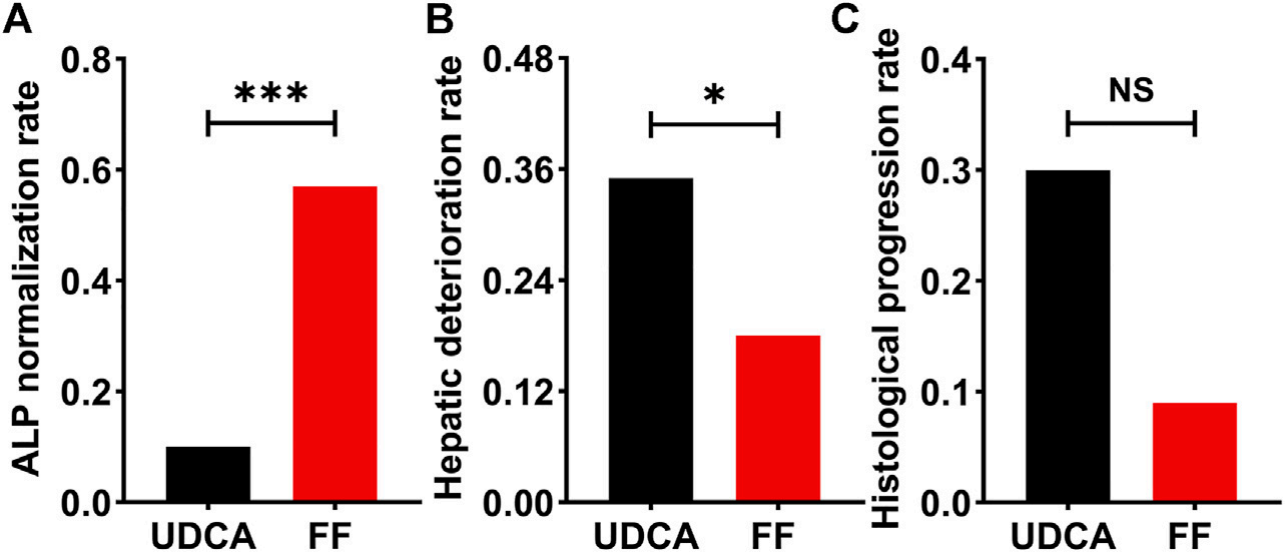
The incidence of primary and secondary outcomes in UDCA-refractory PBC patients treated with UDCA + FF (the FF group) or UDCA monotherapy (the UDCA group). **(A)** ALP normalization rate **(B)** Hepatic deterioration rate **(C)** Histological progression rate. Data was analyzed by the chi-squared test. Abbreviations: UDCA, ursodeoxycholic acid; FF, fenofibrate.

The primary outcome was obtained in 57% of additional fenofibrate-treated cases versus only 10% of UDCA-treated cases (*p* < 0.001) (Figure 8). Univariate analysis of factors related to the biochemical response to fenofibrate showed that six parameters were significantly linked to biochemical response, namely, ALP (*p* = 0.031), albumin (ALB) (*p* = 0.045), TBIL (*p* < 0.001), CRE (*p* = 0.003), eGFR (*p* = 0.003), and cirrhosis (*p* = 0.020) at baseline (Table 4). As shown in Table 4, multivariate analysis incorporating all variables meeting *p* values < 0.05 revealed that the only independent parameter associated with biochemical response to fenofibrate was baseline serum TBIL levels (OR: 0.429; CI: 0.216–0.850; *p* = 0.015). Compared to UDCA-treated cases, fenofibrate-treated cases reported a significantly lower prevalence of hepatic deterioration by study end (35% vs. 18%; *p* = 0.024). Thirty-three patients treated with fenofibrate and twenty patients treated with UDCA underwent at least two liver biopsies at average intervals of 34 (range, 12–84) months and 37 (range, 12–84) months, respectively. Histological progression rates were lower in fenofibrate-treated cases than in UDCA-treated cases, however this difference was not statistically significant (9% vs. 30%; *p* = 0.112) (Figure 8). W\We also provide images of liver biopsy of one patient before and after combination therapy. (Supplementary Figure S11).

**TABLE 4:** Baseline characteristics of patients with UDCA-refractory primary biliary cholangitis treated with “UDCA + FF” between “ALP normalization group” and “ALP non-normalization group”.

Characteristic	ALP normalization	ALP non-normalization	^a^p value	^b^p value	Or (95%CI)
	N = 50	N = 38			
Age (mean)	52 ± 10	48 ± 9	*p* = 0.073		
Female (n, %)	38 (78)	33 (85)	*p* = 0.404		
ALP×ULN	2.3 (2.0–3.0)	2.7 (2.2–3.8)	*p* = 0.031		
GGT×ULN	6.6 (3.7–12.8)	8.4 (6.5–14.6)	*p* = 0.128		
ALT×ULN	1.5 (1.1–2.1)	1.8 (1.3–2.5)	*p* = 0.226		
AST×ULN	1.6 (1.3–2.5)	2.2 (1.5–2.8)	*p* = 0.085		
ALB×LLN	1.1 (1.0–1.1)	1.0 (0.9–1.1)	*p* = 0.045		
Tbil×ULN	0.9 (0.6–1.3)	1.3 (0.9–2.7)	*p* < 0.001	*p* = 0.015	0.429 (0.216–0.850)
IgG×ULN	0.80 (0.69–0.96)	0.75 (0.72–0.86)	*p* = 0.788		
IgM×ULN	1.15 (0.75–1.78)	1.3 (0.8–1.8)	*p* = 0.255		
TG×LLN	1.0 (0.7–1.4)	1.1 (0.8–1.8)	*p* = 0.074		
BU×ULN	0.6 (0.5–0.7)	0.6 (0.5–0.6)	*p* = 0.225		
Scr×ULN	1.0 (0.9–1.1)	0.9 (0.7–1.0)	*p* = 0.003		
eGFR (ml/min/1.73m^2^)	94 (89–101)	100 (95–106)	*p* = 0.003		
Cirrhosis (n, %)	13 (26)	19 (50)	*p* = 0.020		
Fibrosis stage (0–2)/(3–4)	33/14	29/7	*p* = 0.424		

Abbreviations: ALP, alkaline phosphatase; GGT, gamma-glutamyl transferase; ALT, alanine-aminotransferase; AST, aspartate-aminotransferase; ALB, albumin; Tbil, total bilirubin; IgG, immunoglobulin G; IgM, immunoglobulin M; TG, triglyceride; BU, blood urea; Scr, serum creatinine; eGFR, estimated glomerular filtration rate; ULN, upper limit of normal; LLN, lower limit of normal; UDCA, ursodeoxycholic acid; FF, fenofibrate; OR, odds ratio; CI, confidence interval.

a Univariate analysis.

b Multivariate analysis: Logistic regression analysis.

### Biochemical measures

**FIGURE 9:**
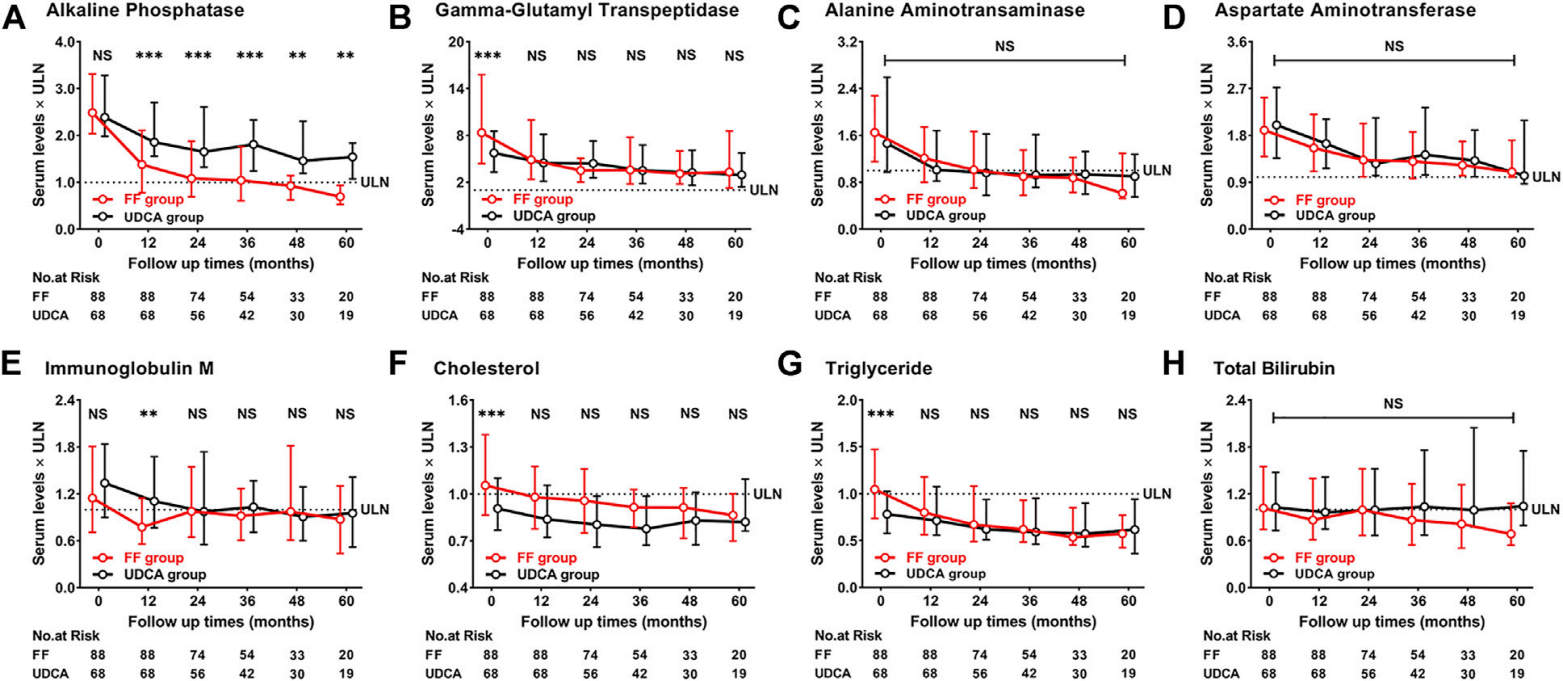
Dynamic changes of parameters with follow-up time **(A)** Alkaline Phosphatase **(B)** Gamma-Glutamyl Transpeptidase **(C)** Alanine Aminotransferase **(D)** Aspartate Aminotransferase **(E)** Immunoglobulin M **(F)** Cholesterol **(G)** Triglyceride **(H)** Total Bilirubin. Group, patients with UDCA-refractory PBC treated with UDCA + FF (the FF group) or UDCA (the UDCA group). Shown are the median values and interquartile ranges at each follow-up visit. Data was compared with the Mann–Whitney *U* test. Abbreviations: UDCA, ursodeoxycholic acid; FF, fenofibrate; ULN, upper limit of the normal range; LLN, lower limit of the normal range.

Figure 9 shows the dynamic changes in ALP, GGT, ALT, AST, IgM, CHO, TG and TBIL. Biochemical characteristics of patients with UDCA-refractory PBC between “the FF group” and “the UDCA group” after 1 year of treatment were shown in Supplementary Table S2. At 60 months, the level of ALP decreased 73% from baseline in “the UDCA group” and 34% in “the UDCA + FF group” (*p* < 0.001, both) (Supplementary Table S3). Similar reductions were found in TG and CHO in “the UDCA + FF group” (*p* < 0.050, all) (Supplementary Table S3). The median serum TBIL level in “the UDCA + FF group” was observed to be 32% lower than baseline at 60 months (*p* = 0.047) (Supplementary Table S3). ALT and AST decreased progressively in both groups during follow-up (*p* < 0.05, all) (Supplementary Table S3). Compared to “the UDCA group”, significantly lower ALP level was observed in “the UDCA + FF group” during follow-up. No significant differences in TBIL, ALT, AST, TG, CHO were found between the groups (Supplementary Table S3). Elevated ALP levels were observed in five patients after stopping fenofibrate but not UDCA for 0.25–3 months, and four patients reached normal values after resuming fenofibrate therapy.

### Safety of additional fenofibrate

**FIGURE 10:**
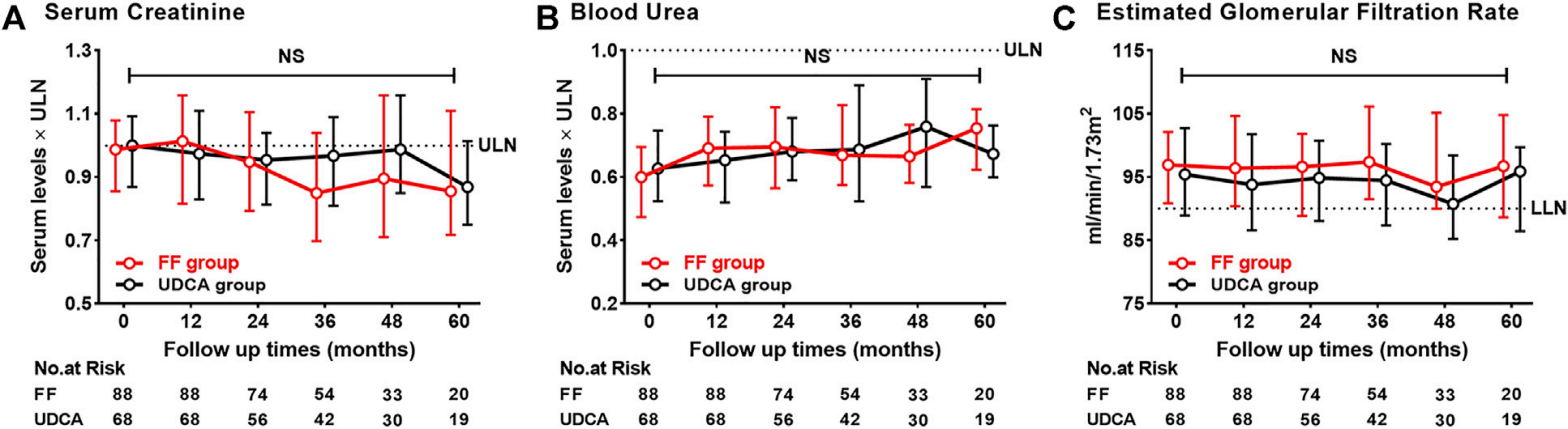
Dynamic changes of parameters with follow-up time **(A)** Serum Creatinine **(B)** Blood Urea **(C)** Estimated Glomerular Filtration Rate. Group, patients with UDCA-refractory PBC treated with UDCA + FF (the FF group) or UDCA (the UDCA group). Shown are the median values and interquartile ranges at each follow-up visit. Data was compared with the Mann–Whitney *U* test. Abbreviations: UDCA, ursodeoxycholic acid; FF, fenofibrate; ULN, upper limit of the normal range; LLN, lower limit of the normal range.

Figure 10 shows that BU, CRE, and eGFR remained stable within the normal range in two groups. Adverse events were reported in Supplementary Table S4. Three patients discontinued use of fenofibrate within 1 month: two patients experienced allergic reactions, and one patient suffered from increased fatigue. Both adverse effects resolved after stopping fenofibrate. Eight patients experienced self-limiting nausea, abdominal pain and bloating in the first 3 months of treatment. Five patients were found to have elevated transaminase levels (ALT or AST, 5–7× ULN) at 12–24 months of treatment with fenofibrate add-on therapy, but ALT levels gradually decreased with continued fenofibrate therapy under monthly monitoring. Four of cirrhotic cases treated with fenofibrate add-on therapy and four of cirrhotic cases treated with UDCA monotherapy experienced severe progression of TBIL levels (>100 mmol/L) (13% vs. 15%; *p* = 1.000). One patient with Child–Pugh B cirrhosis at baseline, who progressed to Child–Pugh C cirrhosis at 96 months of follow-up, developed renal deterioration with an eGFR of 28 ml/min/1.73 m^2^. No severe adverse events were identified in other patients treated with fenofibrate for more than 12 months.

## Discussion

PBC is a chronic intrahepatic cholestatic disease characterized by a progressive nonsuppurative inflammatory reaction of the intrahepatic bile ducts (Kumagi et al., 2010). UDCA is currently the first-line agent for the treatment of PBC. However, some patients could not benefit from UDCA treatment. There is a significant reduction in long-term survival for UDCA-refractory PBC patients, which is a problem for clinical treatment. Our meta-analysis included nine publications with a total of 395 patients, showing that combination therapy was more effective than UDCA monotherapy in decreasing biochemical parameters, including ALP, GGT, IgM, and TG. However, the occurrence of pruritus and adverse events was slightly higher with combination therapy than with UDCA monotherapy. Besides, a total of 156 patients were included in our clinical study, finding that fenofibrate add-on therapy significantly improved the ALP normalization rate among UDCA-refractory PBC patients.

Recently, it has been reported that fibrates can be clinically useful in the treatment of hypertriglyceridemia and mixed hyperlipidemia and have anti-inflammatory, antifibrotic, and cholestasis-lowering effects. Fibrates can also ameliorate the biochemical characteristics of patients with PBC (Cancado et al., 2021); Rosenson et al., 2007, but the change in pruritus symptoms was not obvious. Although many observational studies have been published (Cheung et al., 2016; Corpechot et al., 2018; Duan et al., 2018; Ghonem et al., 2020), the mechanism by which fibrates reduce the biochemical markers of cholestasis and whether the application of fibrates can improve the survival rate of patients with these diseases remain unclear. In addition, the sample sizes of relevant reports are small, and the follow-up times are different. Thus, the results do not clearly reflect the efficacy and safety of the combination of UDCA and fibrates.

Fenofibrate, a peroxisome proliferator-activated receptor (PPAR)-α-selective agonist, is commonly used for the treatment of hypercholesterolemia and hypertriglyceridemia (Huang et al., 2008). Some studies have shown that fenofibrate can improve biochemical and immunological parameters in UDCA-refractory PBC patients by inhibiting bile acid production (Ghonem et al., 2020) without an increase in adverse events. It was suggested that PPAR-MDR3-PL may be the main anti-cholestatic mechanism of fenofibrate (Ghonem et al., 2014; Kok et al., 2003). Multidrug resistance protein 3 (MDR3) from the bile duct membrane side of hepatocytes is the main determinant of phospholipid secretion (Ros et al., 2003). PPAR agonists can promote the excretion of phosphatidylcholine in bile by upregulating MDR3, reducing the cytotoxicity of bile salt cells, and inhibiting the formation of bile acids. Studies suggest that PPARα may exert anti-inflammatory effects by counter-regulating interference with proinflammatory transcription factors, such as nuclear factor-κB (NF-κB) (Roglans et al., 2007), signal transduction and activation factors, and other transcription factor pathways to inhibit mRNA and protein expression, thereby reducing p65-mediated gene activation of proinflammatory cytokines (Chen et al., 2014). However, the mechanism of action of UDCA is different. The main function of UDCA is to improve the balance between toxic and nontoxic hydrophobic bile acids and activate the secretion of bile acids, phospholipids, and cholesterol (Poupon, 2012). Therefore, its mechanism of action does not overlap with that of fenofibrate, and the combination of fenofibrate and UDCA may be more effective than UDCA monotherapy.

There is a lack of multicenter RCTs of fenofibrate combined with UDCA in the treatment of refractory PBC. Therefore, the conclusion of a meta-analysis is still needed to provide medical evidence. At present, fenofibrate is recommended for PBC (Supplementary Table S5). However, there are no guideline giving recommendations on the specific dosage or treatment duration. Clinical practitioners generally refer to the clinical scheme for the treatment of hyperlipidemia, i.e., 200 mg/day.

Thus, we used a meta-analysis to evaluate the efficacy of the combination therapy versus UDCA monotherapy by comparing the changes of parameters in UDCA-refractory PBC patients. Meanwhile, we systematically studied the efficacy and safety of long-term combination therapy in UDCA-refractory PBC patients through our retrospective cohort study. By combining these two aspects of work, our integrated analysis supported the effect of combination therapy on improving the biochemical characteristics of UDCA-refractory PBC and suggested the possible role of dose selection of fenofibrate.

However, there are still deficiencies in this study. Although we first proposed that the dose effect of fenofibrate should be considered, there was no relevant clinical grouping in the clinical trial at our center. Second, although the quality of clinical studies included in the meta-analysis was improved compared with that in 2015, RCT studies still accounted for only a small portion of the included studies. Finally, we mainly evaluated the differences in biochemical indices between combination therapy and monotherapy. Due to the lack of histological evaluation over longer periods, we were unable to confirm whether fenofibrate add-on therapy can delay the histological progression of PBC patient. The safety of combined therapy still needs the support of long-term follow-up data, especially the evaluation of liver histology.

Therefore, we expect that high-quality, well-designed and multi-center RCTs with larger sample sizes will be conducted to comprehensively evaluate the long-term efficacy and safety of UDCA-refractory PBC patients using UDCA in combination with fenofibrate. The results of such studies could help guide the clinical use of fenofibrate in the treatment of UDCA-refractory PBC.

## Conclusion

In summary, combination therapy of fenofibrate and UDCA can improve the main serological indices of UDCA-refractory PBC patients. Moreover, the effectiveness and safety of long-term application of combination therapy were shown in our retrospective cohort study. Finally, a larger sample size and longer follow-up are needed to assess the efficacy and safety of the combination of fenofibrate and UDCA and to observe whether liver histology can be improved with this treatment in longer follow-up.

## Data Availability

The original contributions presented in the study are included in the article/Supplementary Material, further inquiries can be directed to the corresponding authors.

## Abbreviations

ALP, alkaline phosphatase; UDCA, ursodeoxycholic acid; FF, fenofibrate; COM, combination therapy; SD, standard deviation; IV, inverse-variance; CI, confidence interval; df, degrees of freedom; GGT, gamma-glutamyl transferase.
