# Supplementary material for: Efficacy and safety of fenofibrate add-on therapy in patients with primary biliary cholangitis refractory to ursodeoxycholic acid: A retrospective study and updated meta-analysis
Source: Front Pharmacol. 2022 Aug 30;13:948362. doi: 10.3389/fphar.2022.948362 (PMC9468667; doi:10.3389/fphar.2022.948362)
Supplement: Supplementary file 5 [file DataSheet2.docx]

**Supplement：**


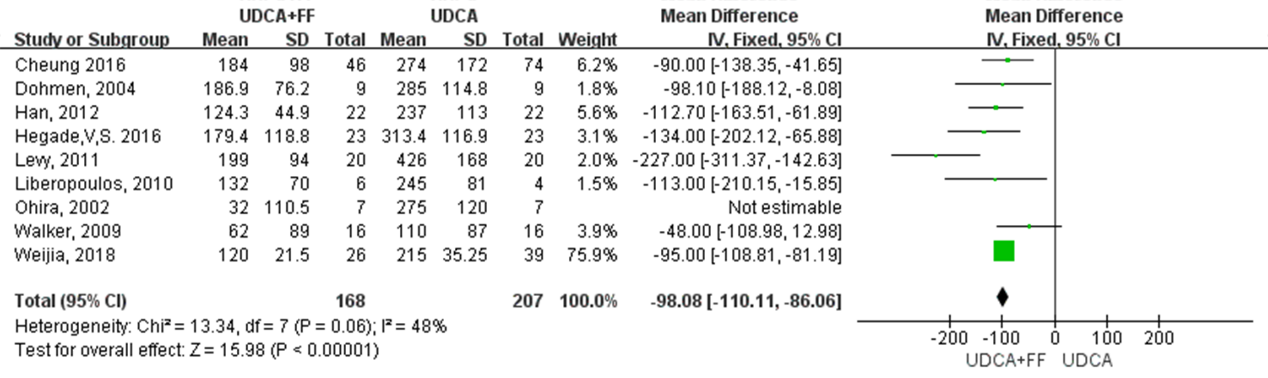


Supplement figure 1. ALP levels in PBC patients treated with UDCA versus UDCA+FF.

Abbreviations: ALP, alkaline phosphatase; UDCA, ursodeoxycholic acid; FF, fenofibrate; SD, standard deviation; IV, inverse-variance; CI, confidence interval; df, degrees of freedom.


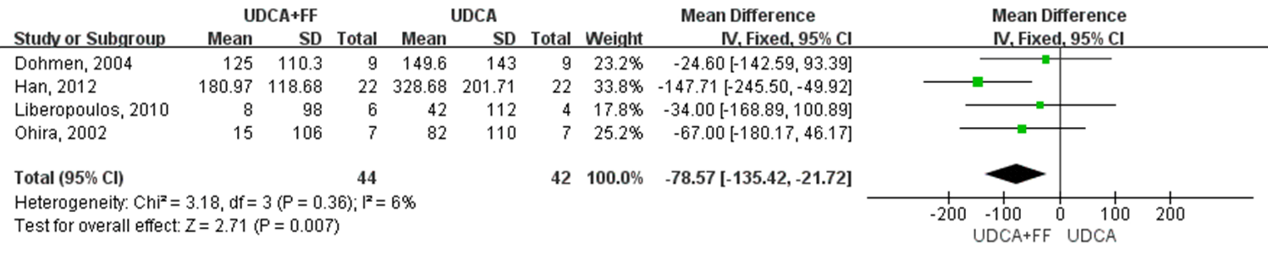


Supplement figure 2. GGT levels in PBC patients treated with UDCA versus UDCA+FF.

Abbreviations: GGT, gamma-glutamyl transferase; UDCA, ursodeoxycholic acid; FF, fenofibrate; SD, standard deviation; IV, inverse-variance; CI, confidence interval; df, degrees of freedom.


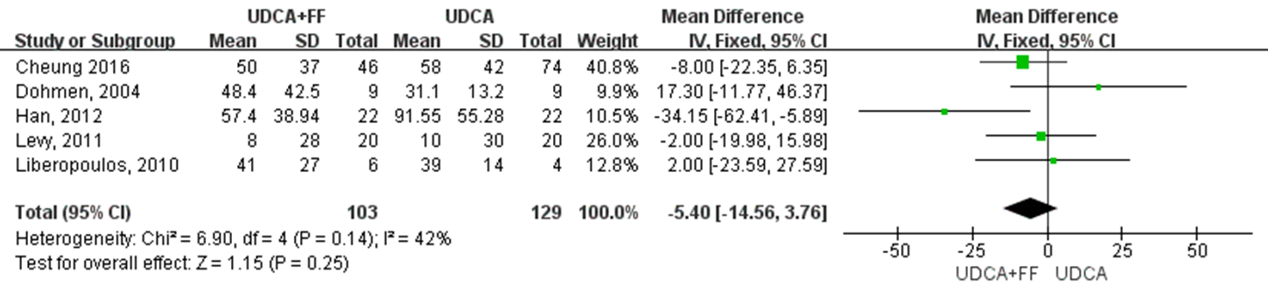


Supplement figure 3. ALT levels in PBC patients treated with UDCA versus UDCA+FF.

Abbreviations: ALT, alanine aminotransferase; UDCA, ursodeoxycholic acid; FF, fenofibrate; SD, standard deviation; IV, inverse-variance; CI, confidence interval; df, degrees of freedom.


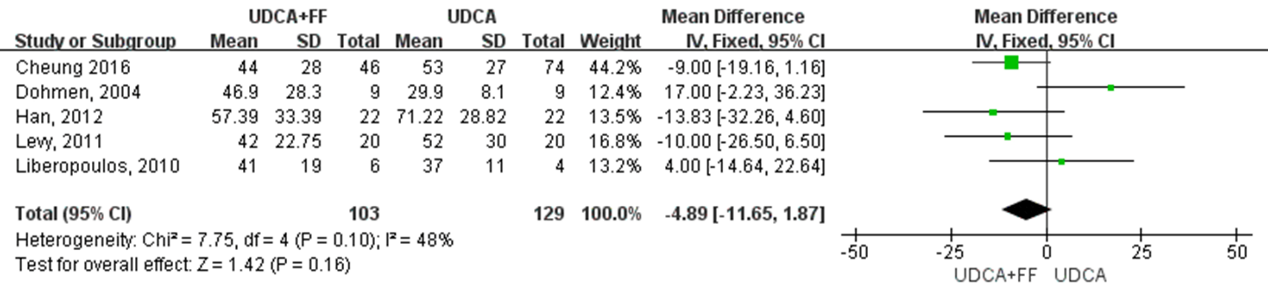


Supplement figure 4. AST levels in PBC patients treated with UDCA versus UDCA+FF.

Abbreviations: AST, aspartate aminotransferase; UDCA, ursodeoxycholic acid; FF, fenofibrate; SD, standard deviation; IV, inverse-variance; CI, confidence interval; df, degrees of freedom.


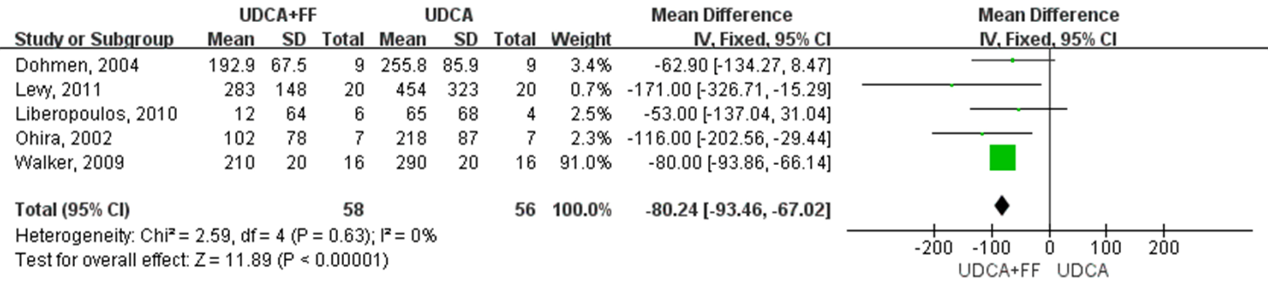


Supplement figure 5. IgM levels in PBC patients treated with UDCA versus UDCA+FF.

Abbreviations: IgM, immunoglobulin M; UDCA, ursodeoxycholic acid; FF, fenofibrate; SD, standard deviation; IV, inverse-variance; CI, confidence interval; df, degrees of freedom.


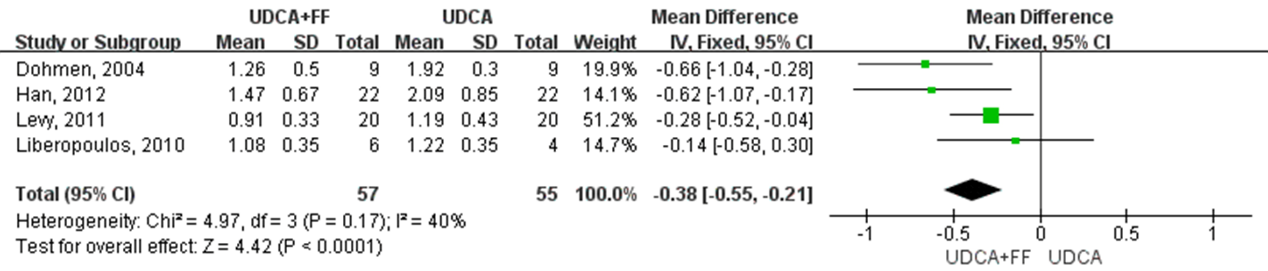


Supplement figure 6. TG levels in PBC patients treated with UDCA versus UDCA+FF.

Abbreviations: TG, triglycerides; UDCA, ursodeoxycholic acid; FF, fenofibrate; SD, standard deviation; IV, inverse-variance; CI, confidence interval; df, degrees of freedom.


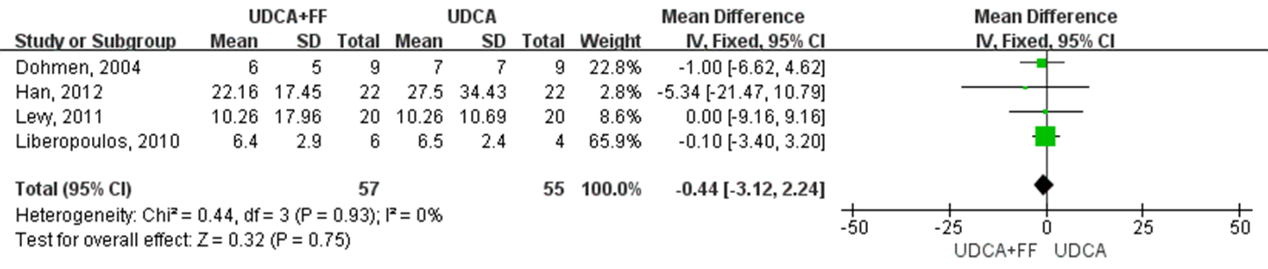


Supplement figure 7. TBIL levels in PBC patients treated with UDCA versus UDCA+FF.

Abbreviations: TBIL, total bilirubin; UDCA, ursodeoxycholic acid; FF, fenofibrate; SD, standard deviation; IV, inverse-variance; CI, confidence interval; df, degrees of freedom.


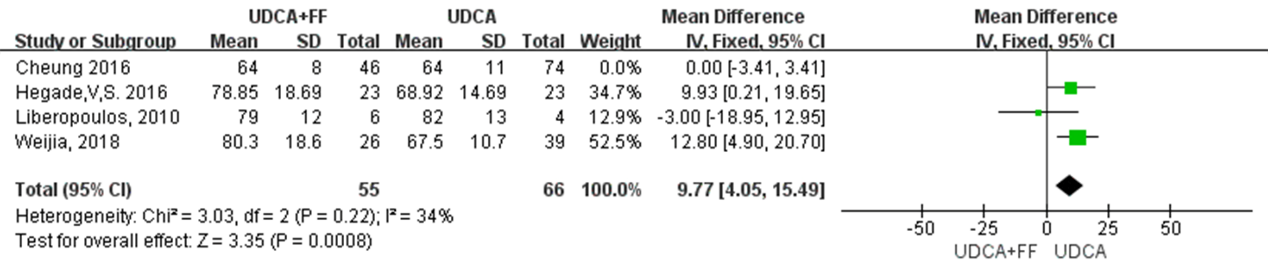


Supplement figure 8. CRE levels in PBC patients treated with UDCA versus UDCA+FF.

Abbreviations: CRE, creatinine; UDCA, ursodeoxycholic acid; FF, fenofibrate; SD, standard deviation; IV, inverse-variance; CI, confidence interval; df, degrees of freedom.


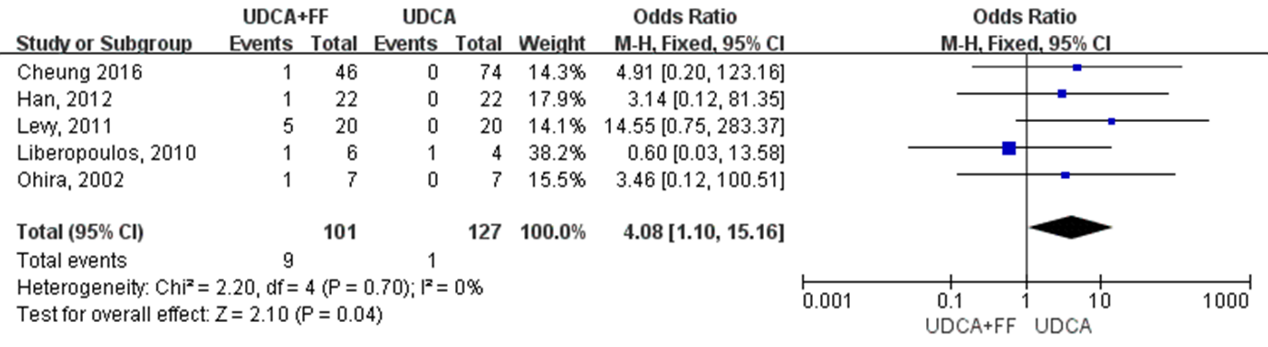


Supplement figure 9. Effects of UDCA versus UDCA+FF on pruritus in patients with PBC.

Abbreviations: UDCA, ursodeoxycholic acid; FF, fenofibrate; M-H, Mantel-Haenszel; CI, confidence interval; df, degrees of freedom.


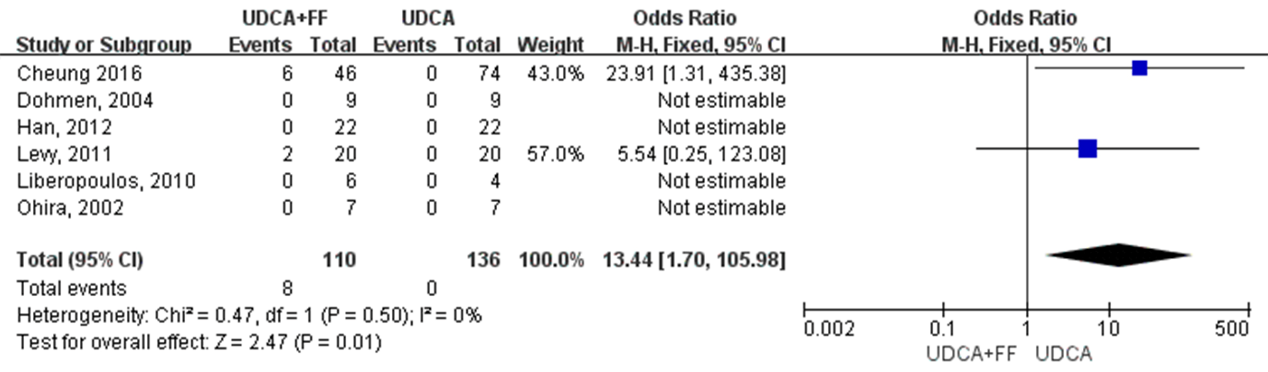


Supplement figure 10. Adverse events in PBC patients treated with UDCA versus UDCA+FF.

Abbreviations: UDCA, ursodeoxycholic acid; FF, fenofibrate; M-H, Mantel-Haenszel; CI, confidence interval; df, degrees of freedom.


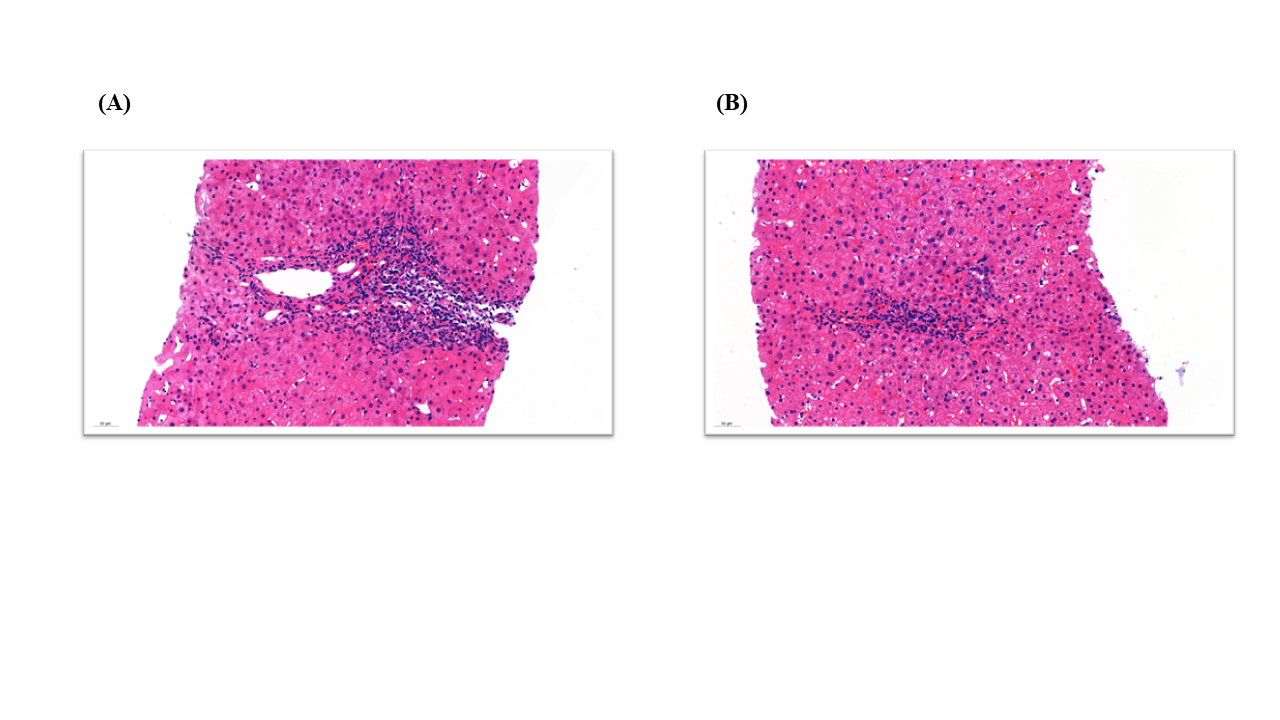


Supplementary figure 11. Liver histology, representative photomicrograph (the scale is 50um) under haematoxylin-eosin staining. A, Baseline biopsy. B, After 5 years of UDCA+FF therapy.
