# Supplementary material for: Efficacy and safety of fenofibrate add-on therapy in patients with primary biliary cholangitis refractory to ursodeoxycholic acid: A retrospective study and updated meta-analysis
Source: Front Pharmacol. 2022 Aug 30;13:948362. doi: 10.3389/fphar.2022.948362 (PMC9468667; doi:10.3389/fphar.2022.948362)
Supplement: Supplementary file 7 [file DataSheet1.docx]

**Supplementary table 1. Fenofibrate-related adverse event**

**Supplementary table 2.** **Biochemical characteristics of patients with UDCA-refractory primary biliary cholangitis between “the FF group” and "the UDCA group" after one year of treatment**

Data are expressed as mean (±SD) or median and IQR. For continuous variables, paired t-test or Mann-Whitney U test was used for normal or non- normal distributed data. Chi-squared test for categorical variables

Abbreviations: ALP, alkaline phosphatase; GGT, gamma-glutamyl transferase; ALT, alanine-aminotransferase; AST, aspartate-aminotransferase; Tbil, total bilirubin; IgM, immunoglobulin M; TG, triglyceride; CHO, cholesterol; BU, blood urea; Scr, serum creatinine; eGFR, estimated glomerular filtration rate; ULN, upper limit of normal; FF, fenofibrate; UDCA, ursodeoxycholic acid; SD, standard deviation; IQR, interquartile range

**Supplementary table 3. Biochemical characteristics of patients with UDCA-refractory primary biliary cholangitis between “the FF group” and "the UDCA group" after five years of treatment**

Data are expressed as mean (±SD) or median and IQR. For continuous variables, paired t-test or Mann-Whitney U test was used for normal or non- normal distributed data. Chi-squared test for categorical variables

Abbreviations: ALP, alkaline phosphatase; GGT, gamma-glutamyl transferase; ALT, alanine-aminotransferase; AST, aspartate-aminotransferase; Tbil, total bilirubin; IgM, immunoglobulin M; TG, triglyceride; CHO, cholesterol; BU, blood urea; Scr, serum creatinine; eGFR, estimated glomerular filtration rate; ULN, upper limit of normal; FF, fenofibrate; UDCA, ursodeoxycholic acid; SD, standard deviation; IQR, interquartile range

**Supplementary table 4. Adverse events in our cohort study**

**Supplementary table 5. Current statements about fibrates from the three continents.**
